# Design and Development of an Automatic Layout Algorithm for Laser GNSS RTK

**DOI:** 10.3390/s24175857

**Published:** 2024-09-09

**Authors:** Jiazhi Tang, Xuan Sun, Xianjian Lu, Jiguang Jia, Shihua Tang

**Affiliations:** 1 College of Mechanic and Control Engineering, Guilin University of Technology, Guilin 541004, China; 2 College of Geomatics and Geoinformation, Guilin University of Technology, Guilin 541004, China

**Keywords:** GNSS RTK, automatic layout, laser, rotation angle

## Abstract

At the current stage, the automation level of GNSS RTK equipment is low, and manual operation leads to decreased accuracy and efficiency in setting out. To address these issues, this paper has designed an algorithm for automatic setting out that resolves the common problem of reduced accuracy in conventional RTK. First, the calculation of the laser rotation center is conducted using relevant parameters to calibrate the instrument’s posture and angle. Then, by analyzing the posture information, the relative position and direction of the instrument to the point to be set out are determined, and the rotation angles in the horizontal and vertical directions are calculated. Following this, the data results are analyzed, and the obtained rotation angles are output to achieve automatic control of the instrument. Finally, a rotating laser composed of servo motors and laser modules is used to control the GNSS RTK equipment to locate the set-out point, thereby determining its position on the ground and displaying it in real-time. Compared to traditional GNSS RTK equipment, the proposed automatic setting out algorithm and the developed GNSS laser RTK equipment reduce the setting out error from 15 mm to 10.3 mm. This reduces the barrier to using GNSS RTK equipment, minimizes human influence, enhances the work efficiency of setting out measurements, and ensures high efficiency and stability under complex conditions.

## 1. Introduction

High-precision positioning is a critical issue for many applications, such as machine control, drones, and mobile mapping. In outdoor environments, the Global Positioning System (GPS [1]) Real-Time Kinematic (RTK [2]) has been proven to be a reliable and efficient tool that can provide centimeter-level [3] positioning accuracy after resolving carrier phase integer ambiguities [4]. Under open-sky conditions, the use of multi-GNSS receivers [5,6] can achieve rapid ambiguity resolution [7,8], but the high cost of multi-GNSS receivers makes many potential applications unfeasible. In the field of engineering measurement applications, pursuing cost-effective and precise solutions continues to be an important aspect of driving project development, especially in today’s era of informational development, where RTK layout [9,10] is gradually becoming the mainstream layout mode.

RTK layout includes the principles of GNSS RTK positioning [11,12] by using GNSS RTK equipment [13,14] to transfer the points and lines from design drawings onto the ground to facilitate construction according to the designs. It is currently the main equipment for layout work, widely used in various engineering constructions. The current RTK layout methods include traditional RTK layout [15], RTK layout with inertial navigation [16,17], and AR image RTK layout [18,19]. Although traditional RTK layout significantly reduces precision errors caused by manual calculations [20] and is easier to operate compared to total station layout, the process requires maintaining a vertical alignment of the RTK antenna phase center [21] with the alignment pole and the points to be laid out to correctly determine their positions. Manual use of the alignment pole [22] for point finding introduces errors due to human operation and results in low RTK layout efficiency. Especially since the real-time positioning error of RTK itself is generally around 1~2 cm, the tip of the centering pole approaches the layout point, and the distance between the pole tip and the layout point is within a few centimeters, the directional error [23] increases significantly as the distance decreases. It will be difficult to control the movement direction and slow the layout speed due to the impact on the layout accuracy. RTK layout with inertial navigation addresses the major issue of maintaining vertical alignment between the alignment pole and the layout points in traditional RTK layout, improving the efficiency of RTK layout to some extent, but it cannot solve the same issues that arise when the pole tip approaches the layout points in ordinary RTK layout. AR image [12] RTK layout represents the latest technology, based on the advantages of inertial navigation RTK layout. It does not require the use of an alignment pole to locate the layout points. Integrating machine vision technology [24,25], it can display the position of the layout points on the image in real time, significantly improving the speed and accuracy of measuring layout points. However, the issue with AR image RTK layout is that to ensure layout accuracy, it still requires moving the alignment pole, which has certain limitations.

**Figure 1:**
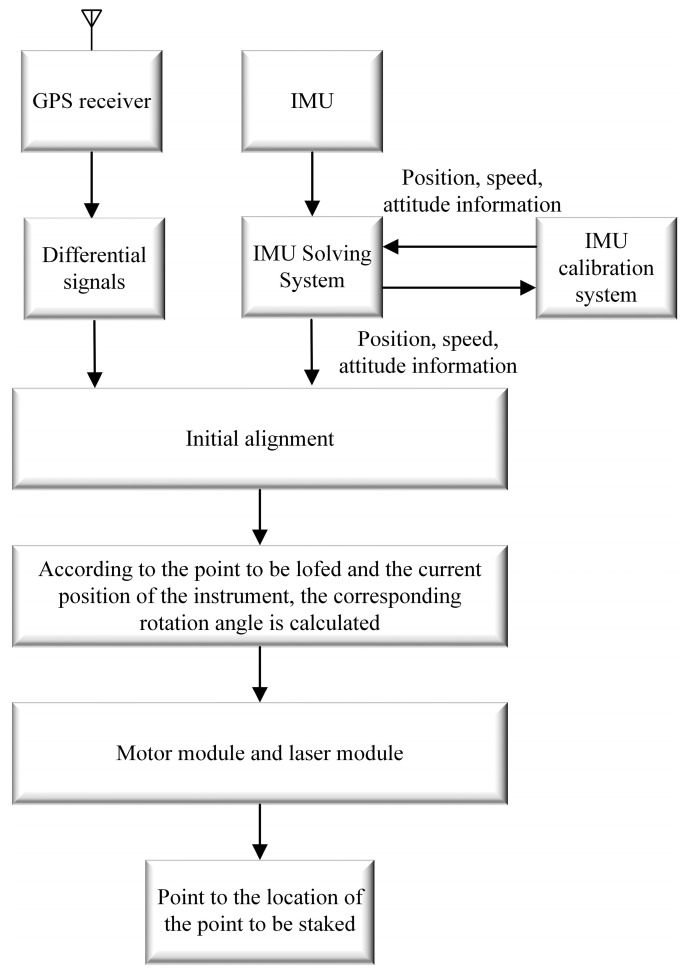
GNSS laser RTK inertial navigation schematic.

**Figure 2:**
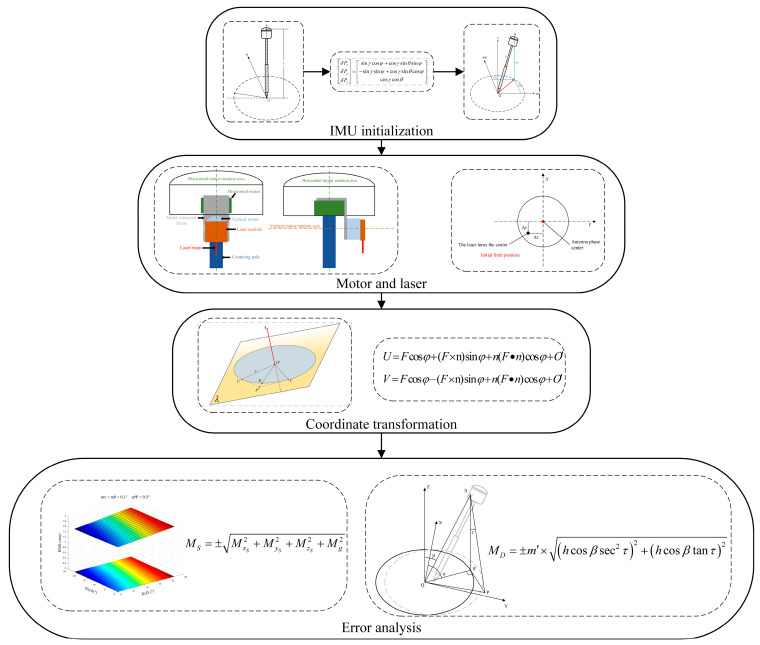
Main content.

To overcome the limitations of the current RTK layout method and meet new societal demands, this paper introduces a high-precision automatic layout computation method that integrates GNSS and inertial navigation systems (Figure 1 and Figure 2). Initially, the method precisely calculates the rotation center of the laser to calibrate the instrument’s orientation and angles, ensuring accurate measurement of the relative position and direction between the instrument and the setting-out points. Subsequently, the calculated rotation angles are converted into commands to drive the laser module via a servo motor, accurately guiding the GNSS RTK equipment to locate and mark the layout points. The innovation of this technique lies in its automation, which significantly reduces human error impacts, achieving rapid and precise marking of layout points, thereby enhancing efficiency and accuracy while simplifying operations. Moreover, this study meticulously records data collected from GNSS RTK devices and servo motor sensors, including positional coordinates and rotation angles, to ensure the accuracy and reliability of the results. This research not only provides practical technological solutions for professionals and non-professionals in the setting-out measurement field but also lays the groundwork for further development and innovation in the domain.

## 2. GNSS Laser RTK Calculation Principle

The laser module is mounted on a vertical servo motor, which is fixed together with a horizontal rotation motor. Two servo motors are installed on the GNSS RTK instrument. The alignment pole is marked with scales to determine the distance from the RTK phase center to the tip of the pole. Using GNSS RTK measurements, the coordinates of the RTK phase center are obtained along with the coordinates and inclination angles of the pole tip measured during calibration. These measurements are used to calculate the coordinates of the laser rotation center, the rotational angles between the vertical and horizontal directions of the point to be laid out, and the calibration direction.

### 2.1. IMU RTK Measurement Principles

Define the GNSS RTK phase center as a point A, the tip of the centering pole as a point O, and the distance from the RTK phase center to the tip of the centering pole as *L*. According to conventional layout, when a fixed solution is obtained by moving the RTK instrument left and right or walking a certain distance, inertial navigation is first initialized in order to convert the inertial heading angle to the geodetic azimuth value.

**Figure 3:**
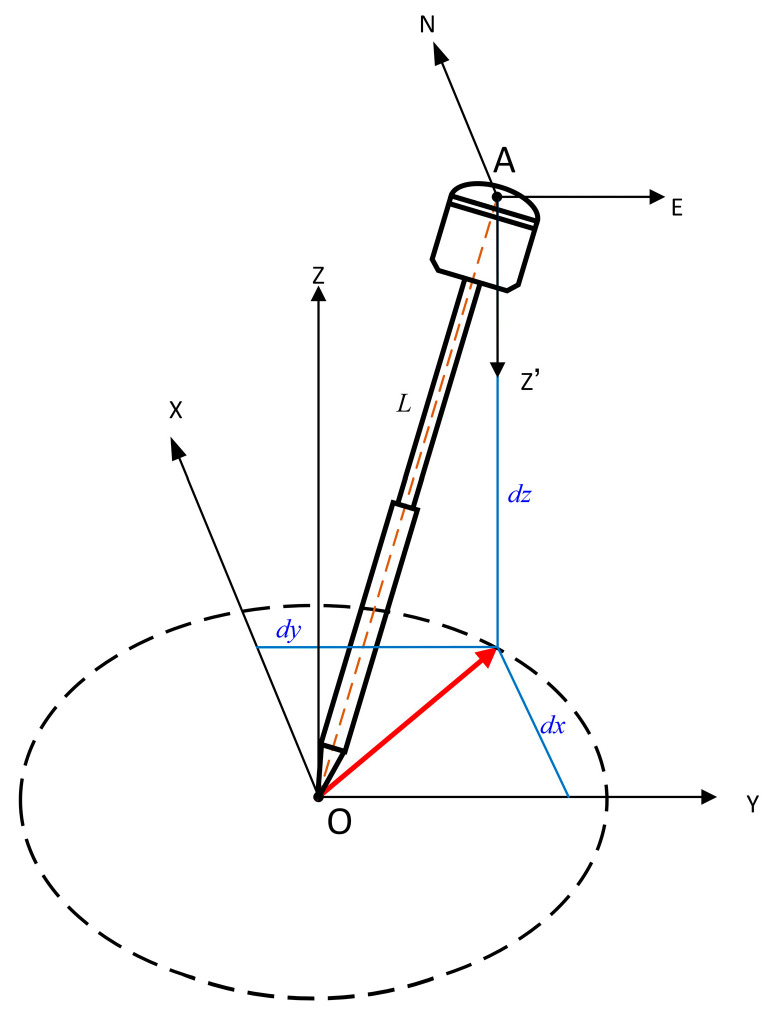
Centering pole inclined posture.

Let the RTK phase center coordinates be $A\left(x_{A},y_{A},z_{A}\right)$. When the RTK is in any inclined state, there is an inclination angle between the centering pole and the ground. The posture of the GNSS RTK equipment is shown in Figure 3. It is necessary to perform a coordinate conversion for the coordinates of the tip of the centering pole.

Assuming the phase center A as the origin of the inertial navigation coordinate system, with the measurement coordinate system being O−XYZ and the Euler angles corresponding to the inertial navigation attitude being roll angle, pitch angle, and yaw angle, represented by symbols γ, θ, and ψ, respectively, their three-axis rotation matrix is as follows:

Rotation around the X-axis:

(1) $$R_{Z}(\psi )=\left[\begin{array}{ccc} \cos\psi & -\sin\psi & 0\\ \sin\psi & \cos\psi & 0\\ 0 & 0 & 1 \end{array}\right]$$

Rotation around the Y-axis:

(2) $$R_{Y}(\theta )=\left[\begin{array}{ccc} \cos & 0 & \sin\theta \\ 0 & 1 & 0\\ -\sin\theta & 0 & \cos\theta \end{array}\right]$$

Rotation around the Z-axis:

(3) $$R_{x}(\gamma )=\left[\begin{array}{ccc} 1 & 0 & 0\\ 0 & \cos\gamma & -\sin\gamma \\ 0 & \sin\gamma & \cos\gamma \end{array}\right]$$

The rotation matrix is:

(4) $$\begin{array}{l} R=R_{Z}\left(\psi \right)R_{Y}\left(\theta \right)R_{X}\left(\gamma \right)\\ =\left[\begin{array}{ccc} \cos\theta \cos\psi & \sin\gamma \sin\theta \cos\psi -\cos\gamma \sin\psi & \cos\gamma \sin\theta \cos\psi +\sin\gamma \sin\psi \\ \cos\theta \sin\psi & \sin\gamma \sin\theta \sin\psi +\cos\gamma \cos\psi & \cos\gamma \sin\theta \sin\psi -\sin\gamma \cos\psi \\ -\sin\theta & \sin\gamma \cos\theta & \cos\gamma \cos\theta \end{array}\right] \end{array}$$

After the initialization of the RTK inertial navigation, the N-axis of the inertial navigation coordinate system corresponds to the X-axis of the measurement coordinate system. In the inertial coordinate system, the unit length coordinate increment from the phase center to the tip of the centering pole is:

(5) $$\begin{array}{l} \left[\begin{array}{c} \Delta x\\ \Delta y\\ \Delta z \end{array}\right]=R\times \left[\begin{array}{c} 0\\ 0\\ 1 \end{array}\right]\\ =\left[\begin{array}{ccc} \cos\theta \cos\psi & \sin\gamma \sin\theta \cos\psi -\cos\gamma \sin\psi & \cos\gamma \sin\theta \cos\psi +\sin\gamma \sin\psi \\ \cos\theta \sin\psi & \sin\gamma \sin\theta \sin\psi +\cos\gamma \cos\psi & \cos\gamma \sin\theta \sin\psi -\sin\gamma \cos\psi \\ -\sin\theta & \sin\gamma \cos\theta & \cos\gamma \cos\theta \end{array}\right]\times \left[\begin{array}{c} 0\\ 0\\ 1 \end{array}\right]\\ =\left[\begin{array}{l} \cos\gamma \sin\theta \cos\psi +\sin\gamma \sin\psi \\ \cos\gamma \sin\theta \sin\psi -\sin\gamma \cos\psi \\ \cos\gamma \cos\theta \end{array}\right] \end{array}$$

Taking into account the conversion relationship between the unit length coordinate increments in the inertial coordinate system and the measurement coordinate system $\left[\delta Px,\delta Py,\delta Pz\right]$, it has:

(6) $$\begin{array}{l} \left[\begin{array}{c} \delta P_{x}\\ \delta P_{y}\\ \delta P_{z} \end{array}\right]=\left[\begin{array}{lll} 1 & 0 & 0\\ 0 & 1 & 0\\ 0 & 0 & -1 \end{array}\right]\times \left[\begin{array}{l} \Delta x\\ \Delta y\\ \Delta z \end{array}\right]=\left[\begin{array}{lll} 1 & 0 & 0\\ 0 & 1 & 0\\ 0 & 0 & -1 \end{array}\right]\times \left[\begin{array}{l} \cos\gamma \sin\theta \cos\psi +\sin\gamma \sin\psi \\ \cos\gamma \sin\theta \sin\psi -\sin\gamma \cos\psi \\ \cos\gamma \cos\theta \end{array}\right]\\ =\left[\begin{array}{l} \cos\gamma \sin\theta \cos\psi +\sin\gamma \sin\psi \\ \cos\gamma \sin\theta \sin\psi -\sin\gamma \cos\psi \\ -\cos\gamma \cos\theta \end{array}\right] \end{array}$$

Let the distance of any point along the direction from the antenna phase center point A to the tip of the centering pole be L. The coordinate increment from the antenna phase center point *A* to the tip of the centering pole point O can be determined as follows:

(7) $$\left\{\begin{array}{l} dx=L\times \delta P_{x}=L\times \left(\sin\gamma \sin\psi +\cos\gamma \sin\theta \cos\psi \right)\\ dy=L\times \delta P_{y}=L\times \left(-\sin\gamma \cos\psi +\cos\gamma \sin\theta \sin\psi \right)\\ dz=L\times \delta P_{z}=-L\cos\gamma \cos\theta \end{array}\right.$$

Thus, the three-dimensional coordinates of a point O can be obtained in real time.

(8) $$\left\{\begin{array}{l} x_{O}=x_{A}+dx\\ y_{O}=y_{A}+dy\\ z_{O}=z_{A}+dz \end{array}\right.$$

### 2.2. Laser Beam and Motor Assembly System Calibration

**Figure 4:**
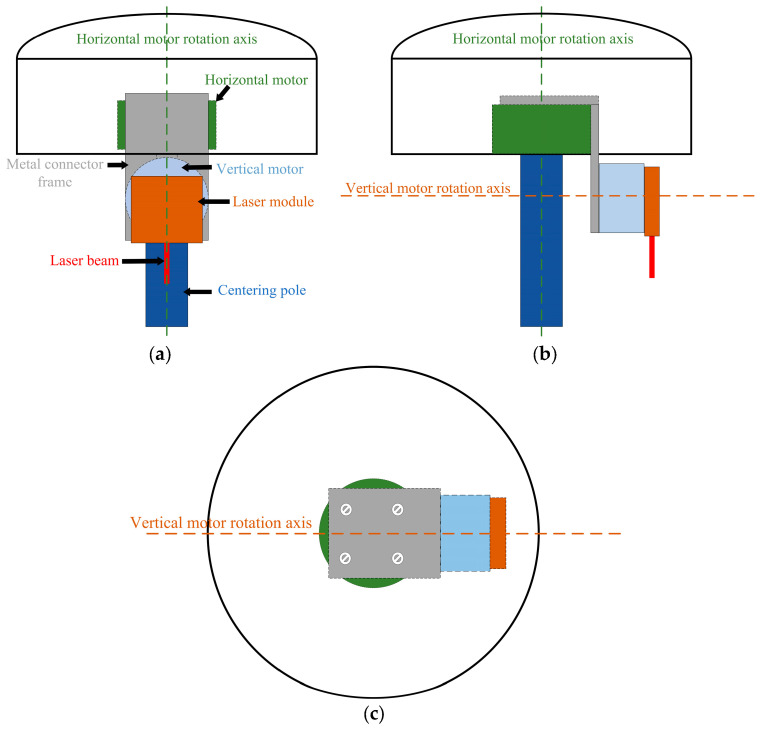
(**a**) Front view of the motor and laser installation method; (**b**) Left view of the motor and laser installation method; (**c**) Top view of the motor and laser installation method.

As shown in Figure 4, the horizontal rotation motor (green area) and the vertical rotation motor (light blue area) are integrated with the laser (orange area) in a specific arrangement. The horizontal motor is integrated onto the GNSS RTK instrument, with its rotation axis aligned with the alignment pole axis. The vertical rotation motor is attached below the horizontal motor using a mounting bracket, with its rotation axis perpendicular to the horizontal rotation axis. The laser device is mounted on the vertical rotation motor, set to emit in a direction perpendicular to the vertical rotation axis, ensuring that the direction of the laser emission is parallel to the axis of the alignment pole.

**Figure 5:**
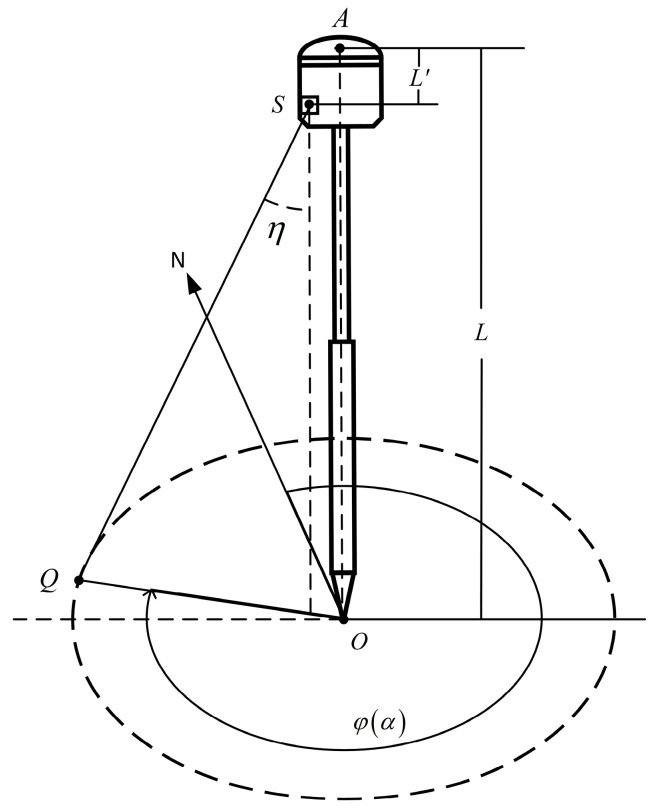
System in plumb state.

After initial alignment [26] is completed, the alignment pole is positioned vertically, putting the system in a plumb state (Figure 5). The laser is directed to align with a known coordinate point $Q\left(x_{Q},y_{Q},z_{Q}\right)$ on the ground. The real-time ground coordinates of the phase center point *A* and the tip of the alignment pole *O* are $\left(x_{A},y_{A},z_{A}\right)$ and $\left(x_{O},y_{O},z_{O}\right)$, respectively. At this time, the roll angle and pitch angle are close to zero, and the heading angle is ψ. *OA* is the length from the phase center point *A* to the ground, and $L^{\prime }$ is the distance from the phase center point *A* along the direction of the alignment pole tip to the laser rotation point *S*.

Since the rotational range of 0 to 180 degrees for the horizontal rotation motor sufficiently meets the practical application needs during the overall structural design of the device, it is specified that when the internal motor of the instrument rotates within this designated angle range, the real-time coordinates of the laser rotation point are $S=\left(x_{S},y_{S},z_{S}\right)$, and the yaw angle rotation matrix is:

(9) $$R_{Z}(\psi )=\left[\begin{array}{ccc} \cos\psi & -\sin\psi & 0\\ \sin\psi & \cos\psi & 0\\ 0 & 0 & 1 \end{array}\right]$$

**Figure 6:**
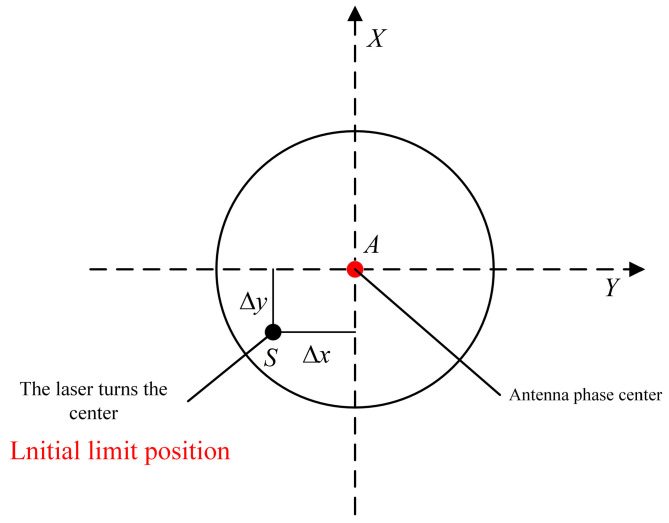
Top view of the center of laser rotation with RTK phase center in plumb case.

In Figure 6, Δx and Δy are the offsets of the laser rotation point S from the RTK phase center point *A* when the instrument is in its internal limit position. The length of $L^{\prime }$ can be determined from the positional relationship between point *S* and point *A*.

By substituting into the rotation matrix $R_{Z}(\psi )$, the three-dimensional coordinates of the laser rotation point $S=\left(x_{S},y_{S},z_{S}\right)$ can be obtained:

(10) $$\left[\begin{array}{c} x_{S}\\ y_{S}\\ z_{S} \end{array}\right]=R_{Z}(\psi )\times \left[\begin{array}{c} x_{A}+\Delta x\\ y_{A}+\Delta y\\ z_{A}-L^{\prime } \end{array}\right]=\left|\begin{array}{ccc} \cos\psi & -\sin\psi & 0\\ \sin\psi & \cos\psi & 0\\ 0 & 0 & 1 \end{array}\right|\times \left[\begin{array}{c} x_{A}+\Delta x\\ y_{A}+\Delta y\\ z_{A}-L^{\prime } \end{array}\right]$$

The azimuth angle of *OQ* can be derived as follows:

(11) $$\alpha_{OQ}=\arctan\left[\frac{y_{Q}-y_{O}}{x_{Q}-x_{O}}\right]$$

(12) $$S_{OQ}=\sqrt{{\left(x_{Q}-x_{O}\right)}^{2}+{\left(y_{Q}-y_{O}\right)}^{2}}$$

The angle between the laser direction and the vertical line is expressed as follows:

(13) $$\eta =\arctan\left(\frac{S_{OQ}}{L-L^{\prime }}\right)$$

At this time, the angles of the horizontal and vertical motors are set according to the aforementioned azimuth and tilt angles. The rotation direction of the laser, using the vertical motor’s axis as a reference, is positive when rotating clockwise and negative when rotating counterclockwise. Considering that the layout point is generally within a few centimeters from the centering pole, the angle of the vertical motor relative to the horizontal motor can typically be controlled within a range of about ±10° to meet practical application needs effectively.

Theoretically, larger angles could be used, but considering that in actual staking work, the centering pole needs to be as close as possible to the stakeout point, and the staking accuracy decreases as the distance between the centering pole and the stakeout point increases, an angle of ±10° is already sufficient given that the centering pole is usually around 1.8 m.

### 2.3. The Angle of Inclination of the Centering Rod in Any State

**Figure 7:**
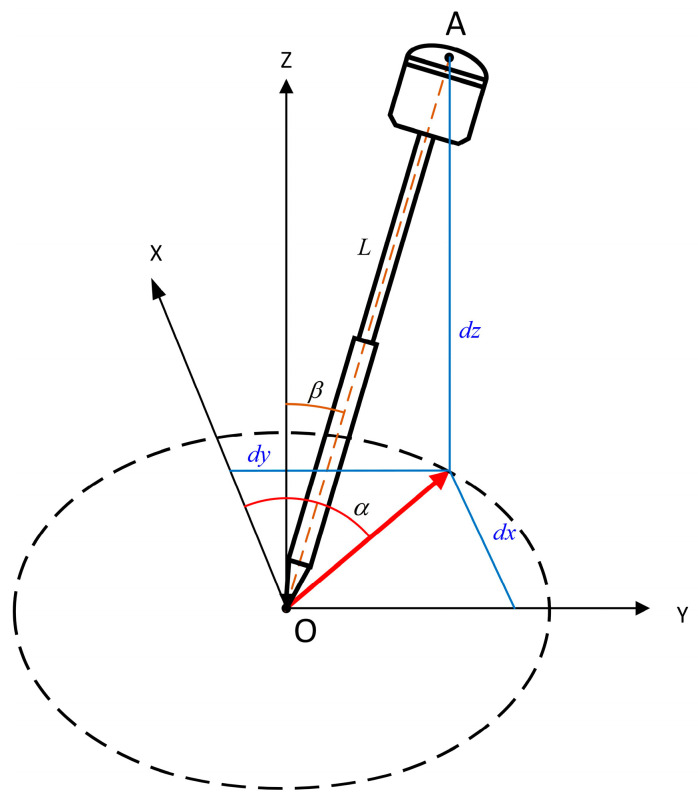
Schematic diagram of the pole tip inclination angle.

Based on the coordinates of the tip of the alignment pole and the instrument’s roll, pitch, and heading angles, the inclination of the alignment pole relative to the ground can be calculated (Figure 7).

From Equation (5), the correction amounts for unit length coordinates in three axes can be obtained. Multiplying these three unit length coordinate corrections by the RTK alignment pole length L yields the projected lengths along the respective axes. Let the inclination angle of the pole be β, and the azimuth angle from the phase center projected onto the ground to the direction of the pole tip be α.

(14) $$\alpha =\arctan\left[\frac{\delta P_{y}}{\delta P_{x}}\right]$$

(15) $$\beta =\arccos\left(\delta P_{z}\right)$$

### 2.4. Principle of Motor Rotation Angle Calculation

When the alignment pole is in a tilted position, the rotation plane of the horizontal motor always aligns with the inclined plane of the RTK phase center; therefore, the normal vector of the horizontal motor’s rotation plane should be the normal vector of the RTK phase center’s inclined plane.

**Figure 8:**
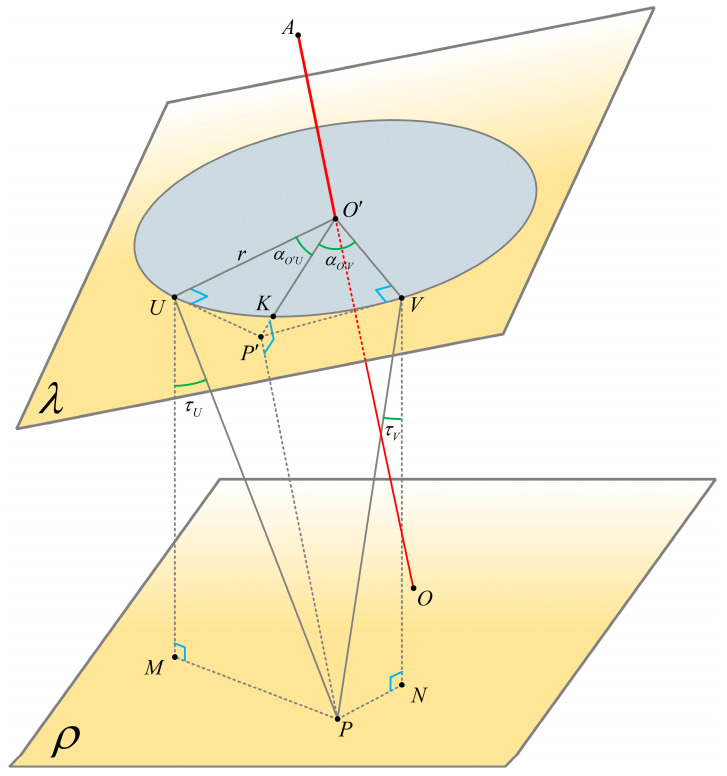
Schematic diagram of motor rotation points.

As shown in Figure 8, the diagram illustrates the motor rotation point relationship:

- Plane ρ is the horizontal plane, and plane λ is an inclined plane.
- *OA* represents the rotation axis of the alignment pole in any state, with its direction vector.
- Point $O^{\prime }$ is the intersection perpendicular to *OA* through the laser rotation point, with a known length *r*.
- A circle with radius r centered at a point $O^{\prime }$ lies on a plane λ.
- Point *P* is the point to be laid out with known specific coordinates; a perpendicular from point *P* to the plane λ intersects at point $P^{\prime }$.
- Point K is the intersection of $O^{\prime }P^{\prime }$ and the laser rotation radius, where $O^{\prime }P^{\prime }$ intersects with $⊙O^{\prime }$ at point *K*.
- Through point $P^{\prime }$, tangents from point $P^{\prime }$ to $⊙O^{\prime }$ are drawn at points *U* and *V*, and perpendiculars from points *U* and *V* to plane ρ intersect at points *M* and *N*, respectively.

The coordinates and orientation of the RTK phase center $A\left(x_{A},y_{A},z_{A}\right)$ can be obtained in real time. The coordinates and orientation of the perpendicular intersection point $O^{\prime }\left(x_{o^{\prime }},y_{o^{\prime }},z_{o^{\prime }}\right)$ with the axis of the alignment pole can also be acquired in real time. By calculating the tip of the alignment pole *O* and the RTK phase center point *A*, the unit vector of *OA* is obtained as $\left(dpx,dpy,dpz\right)$. Therefore, the three-dimensional coordinates of the point $O^{\prime }$ are expressed as follows:

(16) $$\left[\begin{array}{c} X^{\prime }\\ Y^{\prime }\\ Z^{\prime } \end{array}\right]=\left[\begin{array}{c} x_{A}+L^{\prime }*dpx\\ y_{A}+L^{\prime }*dpy\\ z_{A}+L^{\prime }*dpz \end{array}\right]$$

$P\left(x_{P},y_{P},z_{P}\right)$ is the ground point to be laid out. From Equation (8), the coordinates of the point $O\left(x_{O},y_{O},z_{O}\right)$ are known. The equation for plane λ, which passes through $O^{\prime }$ and is perpendicular to the alignment pole:

(17) $$dpx(x-X^{\prime })+dpy\left(y-Y^{\prime }\right)+dpz\left(z-Z^{\prime }\right)=0$$

With the substitutions of A=dpx, B=dpy, and C=dpz, Equation (17) can be written as:

(18) $$A(x-X^{\prime })+B\left(y-Y^{\prime }\right)+C\left(z-Z^{\prime }\right)=0$$

or

(19) $$Ax+By+Cz-\left(AX^{\prime }+BY^{\prime }+CZ^{\prime }\right)=0$$

$D=-\left(AX^{\prime }+BY^{\prime }+CZ^{\prime }\right)$; the above equation can be obtained the following way:

(20) Ax+By+Cz+D=0

The coordinates of the point $P\left(x_{P},y_{P},z_{P}\right)$ on an inclined plane λ, which is perpendicular to the ground point $P^{\prime }=\left(x_{P^{\prime }},y_{P^{\prime }},z_{P^{\prime }}\right)$, are expressed as follows:

(21) $$\left[\begin{array}{c} x_{P^{\prime }}\\ y_{P^{\prime }}\\ z_{P^{\prime }} \end{array}\right]=\left[\begin{array}{c} x_{P}-A(Ax+By+Cz+D)\\ y_{P}-B(Ax+By+Cz+D)\\ z_{P}-C(Ax+By+Cz+D) \end{array}\right]$$

The length of $O^{\prime }P^{\prime }$:

(22) $$S_{O^{\prime }P^{\prime }}=\sqrt{{\left(x_{P^{\prime }}-X^{\prime }\right)}^{2}+{\left(y_{P^{\prime }}-Y^{\prime }\right)}^{2}+{\left(z_{P^{\prime }}-Z^{\prime }\right)}^{2}}$$

(23) $$\vec{O^{\prime }P^{\prime }}=(\Delta x,\Delta y,\Delta z)$$

(24) $$\vec{O^{\prime }K}=\frac{r}{\left|\vec{O^{\prime }P^{\prime }}\right|}\cdot {\left[\Delta x,\Delta y,\Delta z\right]}^{\prime }$$

Calculate the azimuth angle of the horizontal motor rotation:

(25) $$\alpha_{O^{\prime }P^{\prime }}=\arctan\left(\frac{\Delta y}{\Delta x}\right)$$

*U* and *V* are the tangent points on the circular path of the horizontal motor through *P*′. If the laser point is to precisely land on the layout point *P*, the horizontal motor needs to rotate to this position. Here, $P^{\prime }U⊥O^{\prime }U$, $P^{\prime }V⊥O^{\prime }V$, and $∠KO^{\prime }U=∠KO^{\prime }V=\varphi$.

(26) $$\varphi =\arccos\left[\frac{r}{S_{O^{\prime }P^{\prime }}}\right]$$

On this inclined plane, by rotating the vector $\vec{O^{\prime }K}$ by ±φ degrees, the spatial positions of points *U* and *V* where the laser rotation point needs to move can be obtained.

Let the vectors $\vec{O^{\prime }K}=F$ and $\vec{n}=\left(A,B,C\right)$ be the normal vectors of the plane λ. Using Rodrigues’ rotation formula [27]. For space vectors, the coordinates of the two tangent points can be calculated:

(27) $$U=F\cos\varphi +(F\times n)\sin\varphi +n(F•n)\cos\varphi +O^{\prime }$$

(28) $$V=F\cos\varphi -(F\times n)\sin\varphi +n(F•n)\cos\varphi +O^{\prime }$$

where point *U* rotates counterclockwise by θ degrees and point *V* rotates clockwise by θ degrees.

Vectors $D_{1}=U-O^{\prime }$ and $D_{2}=V-O^{\prime }$, according to the coordinates of the tangent points, can have their azimuth angles to the point $O^{\prime }$ calculated. These represent the horizontal rotation angles of the motor.

(29) $$\alpha_{O^{\prime }U}=\arctan\left[\frac{D_{1}(2,1)}{D_{1}(1,1)}\right]$$

(30) $$\alpha_{O^{\prime }V}=\arctan\left[\frac{D_{2}(2,1)}{D_{2}(1,1)}\right]$$

When rotating the motor, it can be set to rotate to the position that has the smallest angular difference from the current motor position (azimuth). If the motor rotates to point *U*, let the vertical motor’s tilt rotation angle be τ, which is equal to the angle between and the unit vector $i=\left(0,0,1\right)$.

(31) $$\vec{UP}=P-U$$

(32) $$\tau_{U}=\arccos\left(\frac{\vec{UP}\cdot i}{\left|\vec{UP}\right|}\right)$$

(33) $$\vec{VP}=P-V$$

(34) $$\tau_{V}=-\arccos\left(\frac{\vec{VP}\cdot i}{\left|\vec{VP}\right|}\right)$$

## 3. Example Verification

Perform data validation for the GNSS Laser RTK fusion algorithm, obtaining RTK attitude data and the coordinates of the point to be laid out in any inclined state to calculate the horizontal and vertical rotation angles of the motor. The steps are as follows:

1. Obtain the RTK phase center coordinates and the coordinates of the point to be laid out based on the RTK attitude, with related data shown in Table 1.

2. After initialization, the IMU attitude (in radians) is expressed as follows:
  γ=0.0872662, θ=0.1523596, ψ=0.9830746

The phase center height is 1.800 m, and the starting position of the laser rotation point relative to the phase center design is (−0.046, 0, −0.058).

3. Coordinates of the pole tip O and point $O^{\prime }$ are shown in Table 2.

4. Center the rod shaft unit vector n.
$$n=\left(A,B,C\right)=\left(0.156363,\;0.077499,\;-0.984654\right)$$
5. Calculate the coordinates of the intersection point $P^{\prime }$ perpendicular to the inclined plane through the ground point *P*; compute the length of $O^{\prime }P^{\prime }$ as well as the azimuth angle $\alpha_{O^{\prime }P^{\prime }}$ of the horizontal motor rotation. Relevant data are shown in Table 3 and Table 4.

6. Coordinates of points U and V are shown in Table 5.

7. The values for the horizontal motor angle and the vertical motor tilt angle are as follows, with relevant data shown below, see Table 6:

## 4. Error Analysis

Considering the effects of inertial navigation on attitude errors, installation errors, etc., the following analysis examines the impact of these errors on the laser rotation point.

### 4.1. Effect of Laser Rotation Point Error

Rotation matrix $R=R_{Z}\left(\psi \right)R_{Z}\left(\theta \right)R_{Z}\left(\gamma \right)$

(35) $$R=\left[\begin{array}{ccc} \cos\theta \cos\psi & \cos\gamma \sin\psi +\sin\gamma \sin\theta \cos\psi & -\sin\gamma \sin\psi +\cos\gamma \sin\theta \cos\psi \\ \cos\theta \sin\psi & -\cos\gamma \cos\psi +\sin\gamma \sin\theta \sin\psi & \;\sin\gamma \cos\psi +\cos\gamma \sin\theta \sin\psi \\ \sin\theta & -\sin\gamma \cos\theta & -\cos\gamma \cos\theta \end{array}\right]$$

The design coordinates of the laser rotation point *S* relative to the phase center $A\left(x_{0},y_{0},z_{0}\right)$ be S(Δx,Δy,Δz), then the real-time three-dimensional coordinates of S:

(36) $$\left[\begin{array}{c} x_{S}\\ y_{S}\\ z_{S} \end{array}\right]=R\times \left[\begin{array}{c} \Delta x\\ \Delta y\\ \Delta z \end{array}\right]\times +\left[\begin{array}{l} x_{0}\\ y_{0}\\ z_{0} \end{array}\right]$$

Expand the above formula to obtain:

(37) $$\left[\begin{array}{c} x_{S}\\ y_{S}\\ z_{S} \end{array}\right]=\left[\begin{array}{ccc} \cos\theta \cos\psi & \cos\gamma \sin\psi +\sin\gamma \sin\theta \cos\psi & -\sin\gamma \sin\psi +\cos\gamma \sin\theta \cos\psi \\ \cos\theta \sin\psi & -\cos\gamma \cos\psi +\sin\gamma \sin\theta \sin\psi & \sin\gamma \cos\psi +\cos\gamma \sin\theta \sin\psi \\ \sin\theta & -\sin\gamma \cos\theta & -\cos\gamma \cos\theta \end{array}\right]\left[\begin{array}{c} \Delta x\\ \Delta y\\ \Delta z \end{array}\right]+\left[\begin{array}{c} x_{0}\\ y_{0}\\ z_{0} \end{array}\right]$$

(38) $$\left\{\begin{array}{l} x_{S}=\Delta x\cos\theta \cos\psi +\Delta y(\cos\gamma \sin\psi +\sin\gamma \sin\theta \cos\psi )+\Delta z(\cos\gamma \sin\theta \cos\psi -\sin\gamma \sin\psi )+x_{0}\\ y_{S}=\Delta x\cos\theta \sin\psi +\Delta y(\sin\gamma \sin\theta \sin\psi -\cos\gamma \cos\psi )+\Delta z(\sin\gamma \cos\psi +\cos\gamma \sin\theta \sin\psi )+y_{0}\\ z_{S}=\Delta x\sin\theta -\Delta y\sin\gamma \cos\theta -\Delta z\cos\gamma \cos\theta +y_{0} \end{array}\right.$$

The impact of the component design coordinates S(Δx,Δy,Δz) along with production and installation errors, set this fixed error to $M_{g}=\pm 1.5\;\mathrm{mm}$. Assuming that the error of the RTK phase center coordinates themselves is not considered. According to the law of error propagation [28], it can be obtained:

(39) $$\begin{array}{cc} dx_{S} & =\left(\Delta y\cos\gamma \sin\theta \cos\psi -\Delta y\sin\gamma \sin\psi -\Delta z\sin\gamma \sin\theta \cos\psi -\Delta z\cos\gamma \sin\psi \right)d\gamma \\ & +\left(\Delta y\sin\gamma \cos\theta \cos\psi +\Delta z\cos\gamma \cos\theta \cos\psi -\Delta x\sin\theta \cos\psi \right)d\theta \\ & +\left(\Delta y\cos\gamma \cos\psi -\Delta y\sin\gamma \sin\theta \sin\psi -\Delta z\sin\gamma \cos\psi \right.\left.-\Delta x\cos\theta \sin\psi -\Delta z\cos\gamma \sin\theta \sin\psi \right)d\psi \end{array}$$

(40) $$\begin{array}{cc} dy_{S} & =\left(\Delta y\sin\gamma \cos\psi +\Delta y\cos\gamma \sin\theta \sin\psi +\Delta z\cos\gamma \cos\psi -\Delta z\sin\gamma \sin\theta \sin\psi \right)d\gamma \\ & +\left(\Delta y\sin\gamma \cos\theta \sin\psi -\Delta x\sin\theta \sin\psi +\Delta z\cos\gamma \cos\theta \sin\psi \right)d\theta \\ & +\left(\Delta x\cos\theta \cos\psi +\Delta y\cos\gamma \sin\psi +\Delta y\sin\gamma \sin\theta \cos\psi -\Delta z\sin\gamma \sin\psi +\Delta z\cos\gamma \sin\theta \cos\psi \right)d\psi \end{array}$$

(41) $$dz_{S}=\left(\Delta z\sin\gamma \cos\theta -\Delta y\cos\gamma \cos\theta \right)d\gamma +\left(\Delta x\cos\theta +\Delta y\sin\gamma \sin\theta +\Delta z\cos\gamma \sin\theta \right)d\theta$$

Assuming the errors of the attitude angles are $m_{\gamma }$, $m_{\theta }$, $m_{\psi }$, and $m_{\gamma }=m_{\theta }=m$, it can be derived that:

(42) $$\begin{array}{cc} M_{x_{S}}^{2} & ={\left(\Delta y\sin\gamma \cos\psi +\Delta y\cos\gamma \sin\theta \sin\psi +\Delta z\cos\gamma \cos\psi -\Delta z\sin\gamma \sin\theta \sin\psi \right)}^{2}m_{\gamma }^{2}\\ & +{\left(\Delta y\sin\gamma \cos\theta \sin\psi -\Delta x\sin\theta \sin\psi +\Delta z\cos\gamma \cos\theta \sin\psi \right)}^{2}m_{\theta }^{2}\\ & +{\left(\Delta x\cos\theta \cos\psi +\Delta y\cos\gamma \sin\psi +\Delta y\sin\gamma \sin\theta \cos\psi -\Delta z\sin\gamma \sin\psi +\Delta z\cos\gamma \sin\theta \cos\psi \right)}^{2}m_{\psi }^{2} \end{array}$$

(43) $$\begin{array}{cc} M_{y_{S}}^{2} & =[{\left(\Delta y\sin\gamma \cos\psi +\Delta y\cos\gamma \sin\theta \sin\psi +\Delta z\cos\gamma \cos\psi -\Delta z\sin\gamma \sin\theta \sin\psi \right)}^{2}\\ & +{\left(\Delta y\sin\gamma \cos\theta \sin\psi -\Delta x\sin\theta \sin\psi +\Delta z\cos\gamma \cos\theta \sin\psi \right)}^{2}]m^{2}\\ & +{\left(\Delta x\cos\theta \cos\psi +\Delta y\cos\gamma \sin\psi +\Delta y\sin\gamma \sin\theta \cos\psi -\Delta z\sin\gamma \sin\psi +\Delta z\cos\gamma \sin\theta \cos\psi \right)}^{2}m_{\psi }^{2} \end{array}$$

Since the impact of the error at the laser rotation point is independent of the magnitude of the heading angle, cosψ can be set to 1 and sinψ to 0. The above formula can be simplified to:

(44) $$\begin{array}{ll} M_{x_{S}}^{2} & =[{\left(\Delta y\cos\gamma \sin\theta -\Delta z\sin\gamma \sin\theta \right)}^{2}+{\left(\Delta y\sin\gamma \cos\theta +\Delta z\cos\gamma \cos\theta -\Delta x\sin\theta \right)}^{2}]m^{2}\\ & +{\left(\Delta y\cos\gamma -\Delta z\sin\gamma \right)}^{2}m_{\psi }^{2} \end{array}$$

(45) $$M_{y_{S}}^{2}=[{\left(\Delta y\sin\gamma +\Delta z\cos\gamma \right)}^{2}m^{2}+{\left(\Delta x\cos\theta +\Delta y\sin\gamma \sin\theta +\Delta z\cos\gamma \sin\theta \right)}^{2}m_{\psi }^{2}$$

(46) $$M_{z_{S}}^{2}=[{\left(\Delta z\sin\gamma \cos\theta -\Delta y\cos\gamma \cos\theta \right)}^{2}+{\left(\Delta x\cos\theta +\Delta y\sin\gamma \sin\theta +\Delta z\cos\gamma \sin\theta \right)}^{2}]m^{2}$$

The coordinate error of the laser rotation point relative to the phase center:

(47) $$M_{S}=\pm \sqrt{M_{x_{S}}^{2}+M_{y_{S}}^{2}+M_{z_{S}}^{2}+M_{g}^{2}}$$

**Figure 9:**
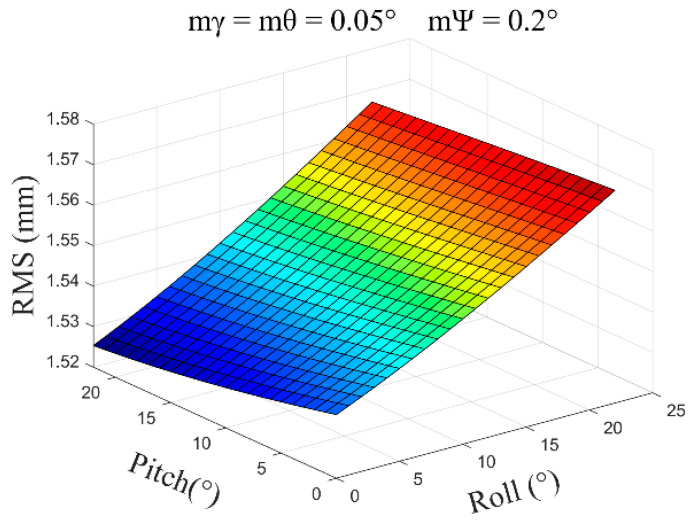
The error distribution chart when the roll angle and pitch angle error are 0.05 degrees and the heading angle error is 0.2 degrees.

**Figure 10:**
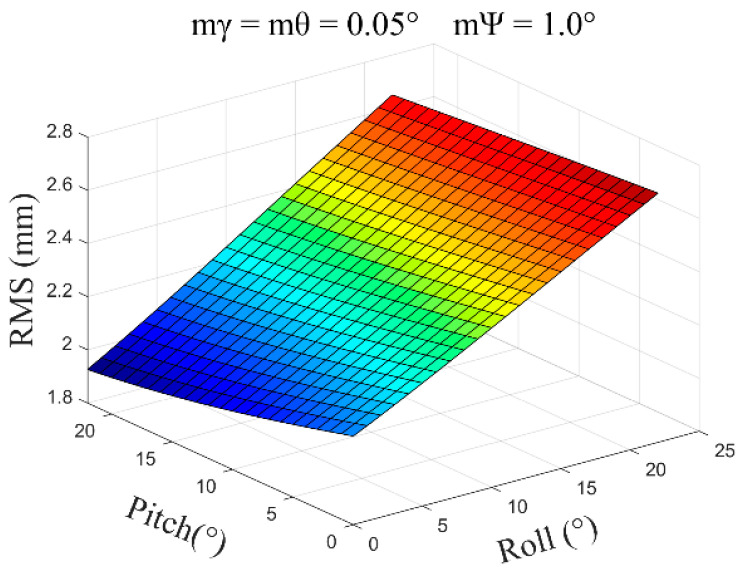
The error distribution chart when the roll angle and pitch angle error are 0.05 degrees and the heading angle error is 1.0 degrees.

**Figure 11:**
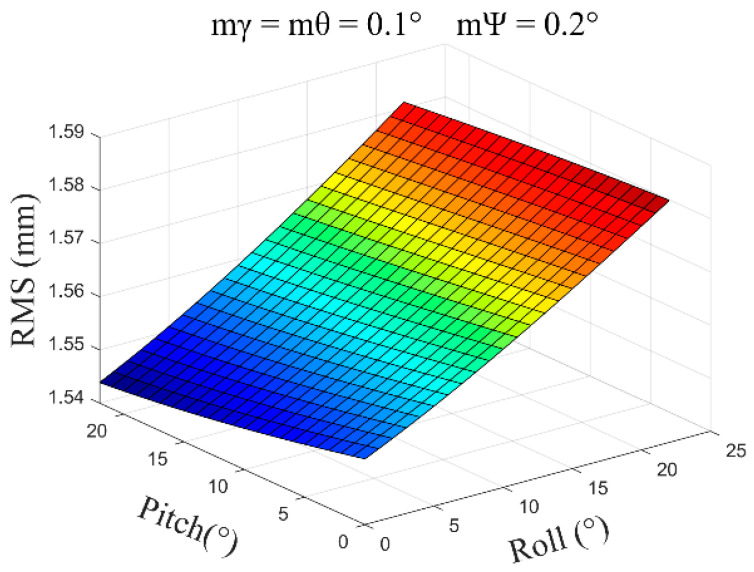
The error distribution chart when the roll angle and pitch angle error are 0.1 degrees and the heading angle error is 0.2 degrees.

**Figure 12:**
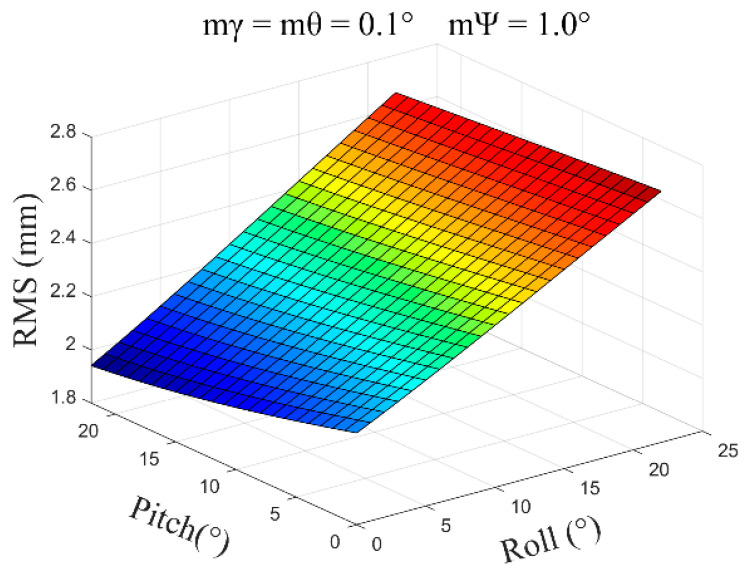
The error distribution chart when the roll angle and pitch angle error are 0.1 degrees and the heading angle error is 1.0 degrees.

Typically, Δx=Δy=0.05m, Δz=0.10m, $m_{\gamma }=m_{\theta }=0.05\sim 0.1{}^{\circ}$, and $m_{\psi }=0.2\sim 1.0{}^{\circ}$ are used for calculations. Some of the calculated results are as follows:

(1) $m_{\gamma }=m_{\theta }=0.05{}^{\circ}$, $m_{\psi }=0.2{}^{\circ}$, β=0°~30°, and the average error obtained is ±1.55 mm (Figure 9).

(2) $m_{\gamma }=m_{\theta }=0.05{}^{\circ}$, $m_{\psi }=1.0{}^{\circ}$, β=0°~30°, and the average error obtained is ±2.35 mm (Figure 10).

(3) $m_{\gamma }=m_{\theta }=0.1{}^{\circ}$, $m_{\psi }=0.2{}^{\circ}$, β=0°~30°, and the average error obtained is ±1.56 mm (Figure 11).

(4) $m_{\gamma }=m_{\theta }=0.1{}^{\circ}$, $m_{\psi }=1.0{}^{\circ}$, β=0°~30°, and the average error obtained is ±2.36 mm (Figure 12).

**Figure 13:**
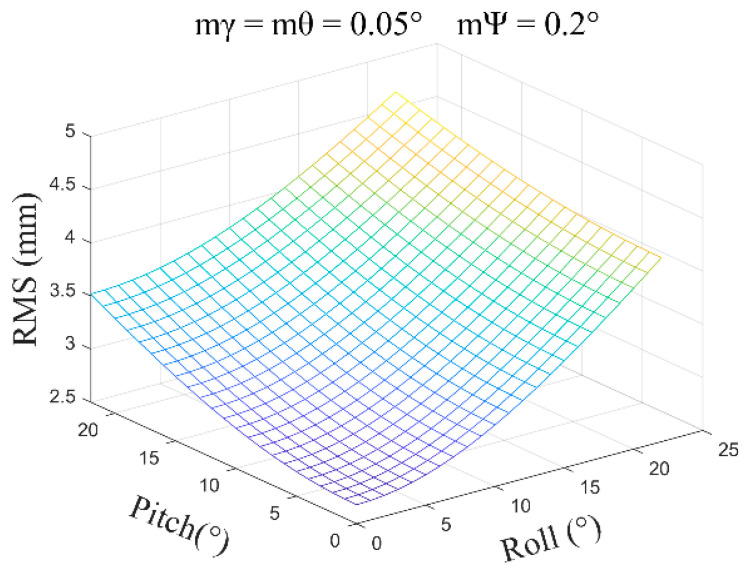
The error distribution chart when the roll angle and pitch angle error are 0.05 degrees and the heading angle error is 0.2 degrees.

**Figure 14:**
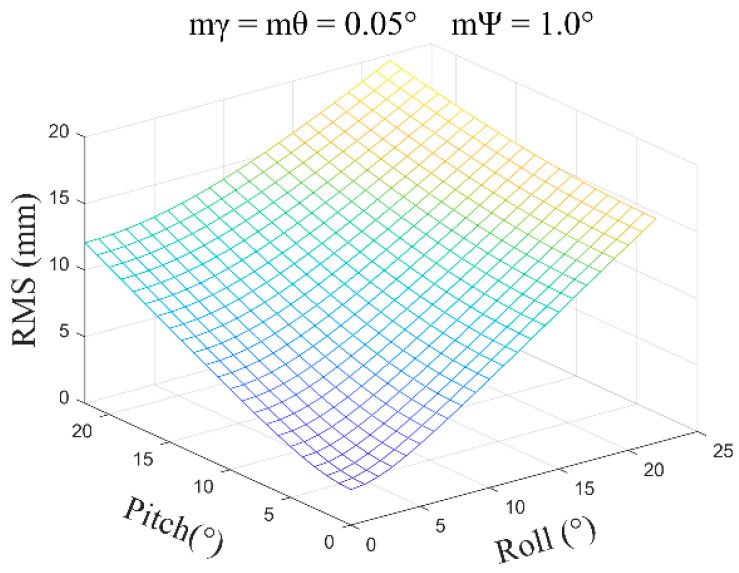
The error distribution chart when the roll angle and pitch angle error are 0.05 degrees and the heading angle error is 1.0 degrees.

**Figure 15:**
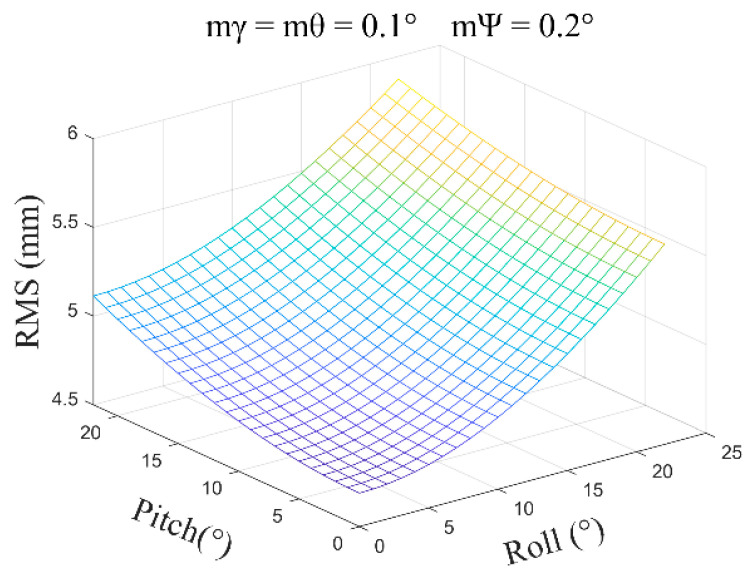
The error distribution chart when the roll angle and pitch angle error are 0.1 degrees and the heading angle error is 0.2 degrees.

**Figure 16:**
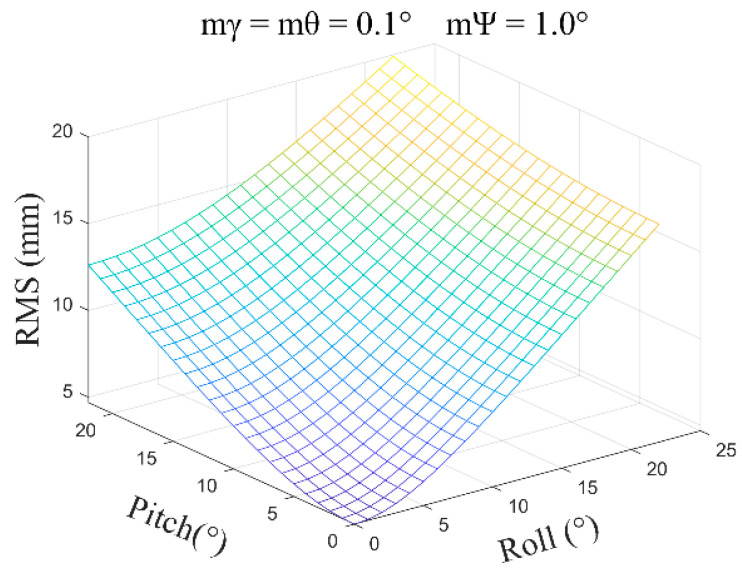
The error distribution chart when the roll angle and pitch angle error are 0.1 degrees and the heading angle error is 1.0 degrees.

When Δx=Δy=0 and Δz=1.8 m, the calculation formula for the measurement error of conventional inertial RTK tilt sampling using a pole can be applied. The error distribution diagram is as follows:

(1) $m_{\gamma }=m_{\theta }=0.1{}^{\circ}$, $m_{\psi }=0.5{}^{\circ}$, β=0°~30°, and the average error obtained is ±3.51 mm (Figure 13).

(2) $m_{\gamma }=m_{\theta }=0.1{}^{\circ}$, $m_{\psi }=1.0{}^{\circ}$, β=0°~30°, and the average error obtained is ±11.40 mm (Figure 14).

(3) $m_{\gamma }=m_{\theta }=0.2{}^{\circ}$, $m_{\psi }=0.5{}^{\circ}$, β=0°~30°, and the average error obtained is ±5.15 mm (Figure 15).

(4) $m_{\gamma }=m_{\theta }=0.2{}^{\circ}$, $m_{\psi }=1.0{}^{\circ}$, β=0°~30°, and the average error obtained is ±12.11 mm (Figure 16).

From the above Figure, it is evident that during conventional inertial RTK layout measurements using an alignment pole, the inclination angle of the pole is generally within 10°. Therefore, the coordinate error $M_{S}$ of the pole tip caused by errors such as attitude angles is approximately ±10 mm, and the error $M_{L}$ due to moving the alignment pole to the point is generally around ±5 mm. Considering that the real-time coordinate error of the RTK phase center is generally within $\pm \left(5\sim 15\right)\;\mathrm{mm}$, with $M_{A}$ taken as ±10 mm, the coordinate error for conventional inertial RTK layout is:

(48) $$M_{P}=\pm \sqrt{M_{A}^{2}+M_{S}^{2}+M_{L}^{2}}=\pm 15\;\mathrm{mm}$$

### 4.2. Effect of Motor Rotation Error

Based on the scheme described in the document and illustrated in the diagram, during laser RTK automatic sampling:

The pole tilt angle is represented by β.

The laser rotation point is denoted as $S\left(x_{S},y_{S},z_{S}\right)$.

The azimuth angle of the horizontal rotation motor is $\alpha_{QS^{\prime }}=\alpha$.

The rotation angle of the vertical rotation motor is τ.

The actual sampling point’s position is denoted as $P\left(x_{P},y_{P},z_{P}\right)$.

The motor rotation angle errors be $m_{\tau }=m_{\gamma }=m^{\prime }$.

$S^{\prime }$ represents the projection of *S* onto the horizontal plane, and its coordinates are $\left(x_{S^{\prime }},y_{S^{\prime }},z_{S^{\prime }}\right)$.

**Figure 17:**
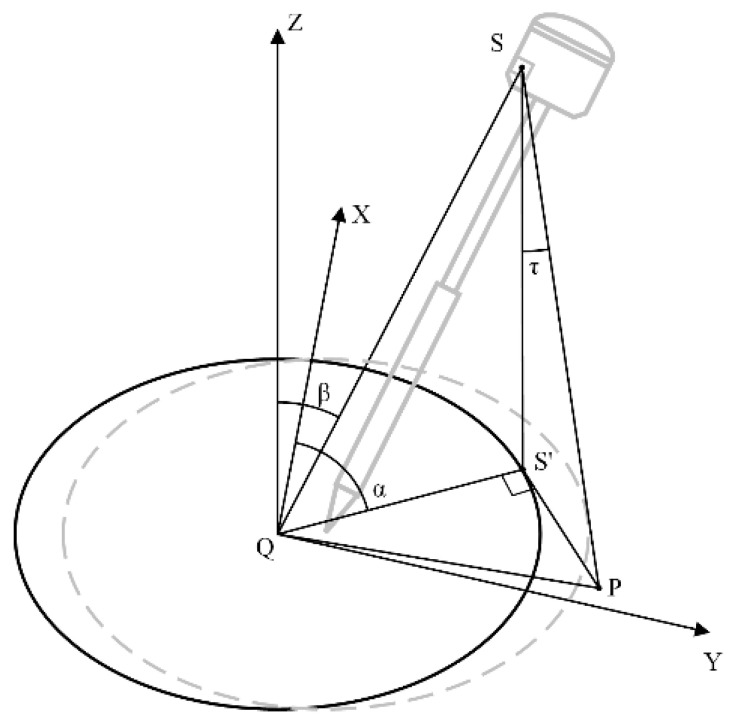
Schematic diagram of motor rotation error.

From Figure 17, that the following can be obtained:

(49) $$\left\{\begin{array}{l} x_{S^{\prime }}=x_{S}\\ y_{S^{\prime }}=y_{S}\\ z_{S^{\prime }}=z_{S}-h\tan\beta \end{array}\right.$$

(50) $$S^{\prime }S=QS\cos\beta =h\cos\beta$$

(51) $$S^{\prime }P=S^{\prime }S\tan\tau =h\cos\beta \tan\tau$$

(52) $$\alpha_{S^{\prime }P}=\alpha_{S^{\prime }Q}-90{}^{\circ}=\left(\alpha_{S^{\prime }Q}\pm 180\right)-90{}^{\circ}=\left\{\begin{array}{l} \alpha +90{}^{\circ}\\ \alpha -270{}^{\circ} \end{array}\right.$$

Since $\sin\left(\alpha +90{}^{\circ}\right)=\sin\left(\alpha -270{}^{\circ}\right)$ and $\cos\left(\alpha +90{}^{\circ}\right)=\cos\left(\alpha -270{}^{\circ}\right)$, the discussion below will only consider the case of $\sin\left(\alpha +90{}^{\circ}\right)$. At the same time, only the impact of errors in the horizontal and vertical motor rotation angles will be considered:

(53) $$\left\{\begin{array}{l} x_{P}=x_{S^{\prime }}+S^{\prime }P\cos\alpha_{MP}=x_{S}-h\cos\beta \tan\tau \sin\alpha \\ y_{P}=y_{S^{\prime }}+S^{\prime }P\sin\alpha_{MP}=y_{S}+h\cos\beta \tan\tau \cos\alpha \\ z_{P}=z_{S^{\prime }}=z_{S}-h\tan\beta \end{array}\right.$$

(54) $$\left\{\begin{array}{l} dx_{P}=dx_{S}-h\cos\beta \sec^{2}\tau \sin\alpha d\tau -h\cos\beta \tan\tau \cos\alpha d\alpha \\ dy_{P}=dy_{S}+h\cos\beta \sec^{2}\tau \cos\alpha d\tau -h\cos\beta \tan\tau \sin\alpha d\alpha \\ dz_{P}=0 \end{array}\right.$$

(55) $$M_{x_{P}}^{2}=M_{x_{S}}^{2}+{\left(h\cos\beta \sec^{2}\tau \sin\alpha \right)}^{2}m_{\tau }^{2}+{\left(h\cos\beta \tan\tau \cos\alpha \right)}^{2}m_{\alpha }^{2}$$

(56) $$M_{y_{P}}^{2}=M_{y_{S}}^{2}+{\left(h\cos\beta \sec^{2}\tau \cos\alpha \right)}^{2}m_{\tau }^{2}+{\left(h\cos\beta \tan\tau \sin\alpha \right)}^{2}m_{\alpha }^{2}$$

The comprehensive error of the laser layout point is:

(57) $$\begin{array}{cc} M_{P}^{2} & =M_{x_{P}}^{2}+M_{y_{P}}^{2}+M_{z_{P}}^{2}\\ & =\left(M_{x_{S}}^{2}+M_{y_{S}}^{2}+M_{z_{S}}^{2}\right)+{\left(h\cos\beta \sec^{2}\tau \sin\alpha \right)}^{2}m_{\tau }^{2}+{\left(h\cos\beta \tan\tau \cos\alpha \right)}^{2}m_{\alpha }^{2}\\ & +{\left(h\cos\beta \sec^{2}\tau \cos\alpha \right)}^{2}m_{\tau }^{2}+{\left(h\cos\beta \tan\tau \sin\alpha \right)}^{2}m_{\alpha }^{2}\\ & =M_{S}^{2}+\left[{\left(h\cos\beta \sec^{2}\tau \right)}^{2}+{\left(h\cos\beta \tan\tau \right)}^{2}\right]{m^{\prime }}^{2}\\ & =M_{S}^{2}+M_{D}^{2} \end{array}$$

The following discussion focuses on the impact of motor rotation errors on the coordinates of the layout points:

(58) $$M_{D}=\pm m^{\prime }\times \sqrt{{\left(h\cos\beta \sec^{2}\tau \right)}^{2}+{\left(h\cos\beta \tan\tau \right)}^{2}}$$

In normal circumstances, the distance from the layout point to the tip of the alignment pole OP can be controlled within approximately 0.1 m to 0.3 m, meaning the vertical motor rotation angle τ is generally within a range of 2°~10°. The inclination angle β is typically within 10°, with motor rotation angle error $m^{\prime }=0.02{}^{\circ}\sim 0.05{}^{\circ}$, and h set at 1.8 m. Based on the above formulas, within a certain range, by considering the relationship between the inclination angle β of the alignment pole and the vertical motor rotation angle τ, the distribution diagram of the layout point position error $M_{D}$ caused by motor rotation errors is shown below.

**Figure 18:**
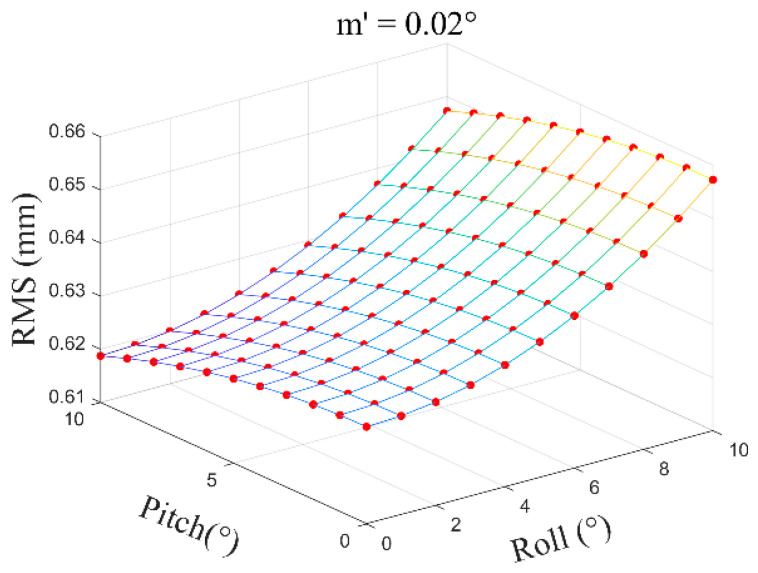
Motor rotation angle error $m^{\prime }=0.02{}^{\circ}$.

**Figure 19:**
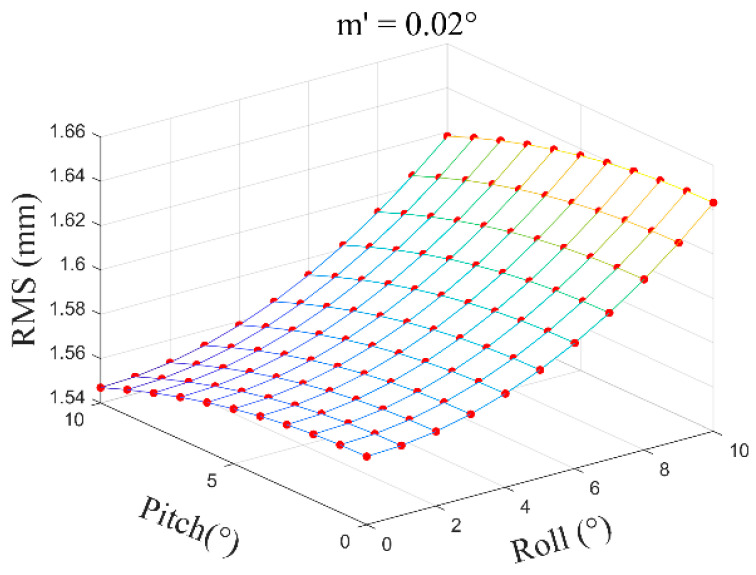
Motor rotation angle error $m^{\prime }=0.05{}^{\circ}$.

(1) With m′ = 0.02°, the average error is ±0.64 mm (Figure 18).

(2) With $m^{\prime }=0.05{}^{\circ}$, the average error is ±1.60 mm (Figure 19).

From the error analysis diagram above, under general conditions, the error of laser-aided automatic layout relative to the antenna phase center can approximately be ±2 mm, and the error caused by motor rotation is ±1.6 mm. Considering the antenna phase center error when using network CORS for RTK measurements, the comprehensive error of the automatic laser inertial RTK layout is:

(59) $$M_{P}=\sqrt{M_{A}^{2}+M_{S}^{2}+M_{D}^{2}}=10.3\;\mathrm{mm}$$

It can be concluded that under existing technical conditions, compared to the phase center, the accuracy of conventional RTK inertial tilt layout using network CORS generally lies within $\pm \left(10\sim 15\right)\;\mathrm{mm}$. Therefore, the laser-aided automatic layout method proposed in this paper exceeds the precision of conventional RTK layout and offers higher work efficiency.

## 5. Experimental Analysis

**Figure 20:**
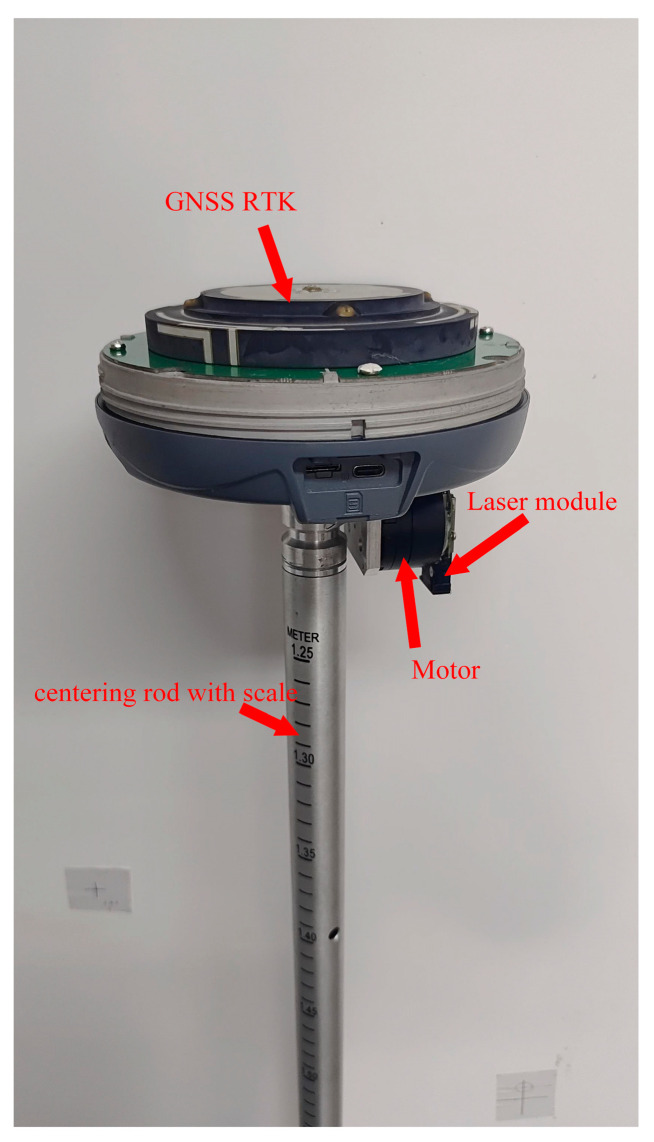
GNSS laser RTK automatic layout engineering prototype.

**Figure 21:**
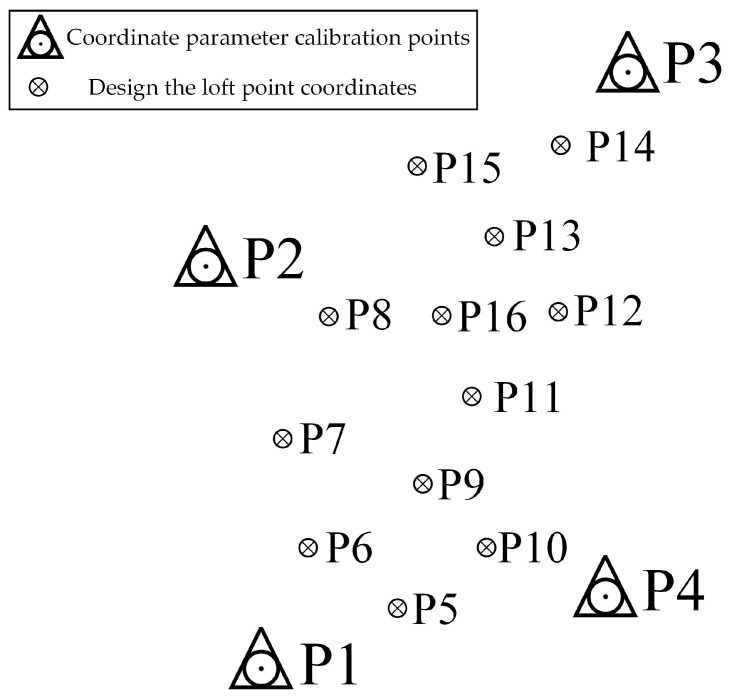
P1–P15 point distribution.

As shown in Figure 20, this experiment uses the laser GNSS RTK prototype developed based on the aforementioned content. Figure 21 shows that four control points P1 to P4 were set up around the test field, and their coordinates were accurately determined using the Leica TS30 total station (Leica, Wetzlar, Germany), which are used for coordinate parameter conversion in RTK. See Table 7.

A set of coordinates for set-out points was designed, initially marked using both conventional RTK and laser automatic RTK. After the set-out is completed, the Leica TS30 total station is used to precisely measure the coordinates of these points and compare them with the designed coordinates to calculate the error m. This provides an objective evaluation of the actual set-out accuracy. The data are shown in Table 8.

(60) $$\left\{\begin{array}{l} \Delta x=X_{design}-X_{lay}\\ \Delta y=Y_{design}-Y_{lay} \end{array}\right.$$

(61) $$\Delta =\sqrt{\Delta x^{2}+\Delta y^{2}}$$

(62) $$m=\pm \sqrt{\frac{\sum \Delta^{2}}{n-1}}$$

### Network Continuous Operational Reference System Was Used for RTK Layout

After confirming the markers using conventional inertial RTK with the Continuous Operational Reference System (CORS) network, the coordinates obtained by precise measurement with the total station are, see Table 9.

After confirming the markers with the laser automatic RTK using the CORS network, the coordinates obtained by precise measurement with the total station are, see Table 10.

Compare with two different RTK layout data, see Table 11.

Due to the measurement errors associated with using the total station, the results calculated from experimental data are slightly larger than the theoretical error values. It can be seen that the laser inertial RTK automatic setting out designed in this paper has higher accuracy compared to conventional inertial RTK layout.

## 6. Conclusions

This paper is based on the fusion algorithm of GNSS RTK and inertial navigation technology, and by utilizing a laser to indicate the location of the points to be laid out, proposes a new theory and implementation method for RTK laser automatic layout. The core idea involves using a new fusion parameter calculation method within traditional RTK layout measurements. It obtains the RTK phase center coordinates of the instrument and the three-dimensional spatial position of the tip of the alignment pole under tilt conditions via inertial navigation offsets. By using mathematical transformations, the specific position of the laser rotation center is determined, and subsequently, the rotational angles between the vertical and horizontal directions of the point to be laid out and the calibration direction are calculated. Finally, two servo motors in the vertical and horizontal directions automatically complete the real-time layout measurement of the designated point, verifying the feasibility of this method. The measurement results meet design requirements, enhancing the positioning accuracy and work efficiency of layout measurements.

The GNSS RTK inertial navigation laser layout method proposed in this paper, compared to current traditional layout, inertial-guided RTK layout, and AR-RTK layout, offers better timeliness and cost-effectiveness, with strong comprehensive surveying capabilities, and is easy to automate. However, there are still some shortcomings in this method, such as accuracy being affected in irregular terrain situations, necessitating further research into corresponding solutions and correction algorithms.

## Figures and Tables

**Table 1:** Coordinates of RTK phase center points *A* and *P* to be staked out.

Point Name	*X* (m)	*Y* (m)	*Z* (m)
*A*	2,797,820.603	430,599.348	150.500
*P*	2,797,820.786	430,599.133	148.706

**Table 2:** Coordinates of the cusp O and $O^{\prime }$ of the centering rod.

Point Name	*X* (m)	*Y* (m)	*Z* (m)
O	2,797,820.888	430,599.488	148.702
$O^{\prime }$	2,797,820.587	430,599.315	150.436

**Table 3:** Coordinates of point $P^{\prime }$.

Point Name	Point Data
$x_{P^{\prime }}\left(m\right)$	2,797,820.517
$y_{P^{\prime }}\left(m\right)$	430,598.999
$z_{p^{\prime }}\left(m\right)$	150.400

**Table 4:** Lengths of $O^{\prime }P^{\prime }$ and angles φ and $\alpha_{O^{\prime }P^{\prime }}$.

Point Name	Point Data
$S_{O^{\prime }P^{\prime }}\left(m\right)$	0.325
$\varphi \left(DMS\right)$	81°51′14.0″
$\alpha_{O^{\prime }P^{\prime }}\left(DMS\right)$	$257{}^{\circ}29^{\prime }36.2^{\prime \prime }$

**Table 5:** Coordinates of tangent points *U* and *V*.

Point Name	*X* (m)	*Y* (m)	*Z* (m)
*U*	2,797,820.560	430,599.355	150.486
*V*	2,797,820.645	430,599.330	150.498

**Table 6:** Rotation angle of horizontal motor and tilt rotation angle of vertical motor.

Location	Horizontal Motor Angle Value	Vertical Motor Angle Value
*U*	$\alpha_{O^{\prime }U}=175{}^{\circ}38^{\prime }22.2^{\prime \prime }$	$\tau_{U}=11{}^{\circ}37^{\prime }16.0^{\prime \prime }$
*V*	$\alpha_{O^{\prime }V}=339{}^{\circ}20^{\prime }50.2^{\prime \prime }$	$\tau_{V}=-11{}^{\circ}37^{\prime }16.0^{\prime \prime }$

**Table 7:** Coordinate parameter calibration points.

Point Name	*X* (m)	*Y* (m)	*Z* (m)
P1	2,798,011.193	431,052.745	154.008
P2	2,798,009.418	431,062.020	154.012
P3	2,798,019.385	431,067.850	154.024
P4	2,798,018.965	431,054.765	154.028

**Table 8:** Design the loft point coordinates.

Point Name	*X* (m)	*Y* (m)
P5	2,798,013.516	431,053.557
P6	2,798,011.330	431,055.442
P7	2,798,011.012	431,058.822
P8	2,798,012.405	431,061.685
P9	2,798,014.713	431,057.192
P10	2,798,016.503	431,054.846
P11	2,798,016.026	431,059.975
P12	2,798,018.573	431,062.162
P13	2,798,016.304	431,063.395
P14	2,798,017.618	431,065.343
P15	2,798,013.902	431,064.398

**Table 9:** Network CORS conventional inertial navigation RTK layout coordinates.

Point Name	*X* (m)	*Y* (m)
P5	2,798,013.520	431,053.574
P6	2,798,011.314	431,055.448
P7	2,798,011.022	431,058.809
P8	2,798,012.416	431,061.697
P9	2,798,014.71	431,057.209
P10	2,798,016.516	431,054.835
P11	2,798,016.01	431,059.971
P12	2,798,018.559	431,062.17
P13	2,798,016.321	431,063.391
P14	2,798,017.626	431,065.327
P15	2,798,013.895	431,064.383

**Table 10:** Network CORS laser inertial navigation RTK automatic layout coordinates.

Point Name	*X* (m)	*Y* (m)
P5	2,798,013.527	431,053.561
P6	2,798,011.323	431,055.451
P7	2,798,011.020	431,058.831
P8	2,798,012.412	431,061.694
P9	2,798,014.704	431,057.184
P10	2,798,016.509	431,054.856
P11	2,798,016.019	431,059.965
P12	2,798,018.565	431,062.17
P13	2,798,016.295	431,063.404
P14	2,798,017.612	431,065.353
P15	2,798,013.909	431,064.408

**Table 11:** Comparison of two different RTK layouts of network CORS.

Number	Conventional Inertial Navigation RTK			Laser Inertial Navigation RTK		
	ΔX (m)	ΔY (m)	Δ (m)	ΔX (m)	ΔY (m)	Δ
1	−0.004	−0.017	0.017	−0.011	−0.004	0.012
2	0.016	−0.006	0.017	0.007	−0.009	0.011
3	−0.01	0.013	0.016	−0.008	−0.009	0.012
4	−0.011	−0.012	0.016	−0.007	−0.009	0.011
5	0.003	−0.017	0.017	0.009	0.008	0.012
6	−0.013	0.011	0.017	−0.006	−0.01	0.012
7	0.016	0.004	0.017	0.007	0.01	0.012
8	0.014	−0.008	0.016	0.008	−0.008	0.011
9	−0.017	0.004	0.018	0.009	−0.009	0.013
10	−0.008	0.016	0.018	0.006	−0.01	0.012
11	0.007	0.015	0.017	−0.007	−0.01	0.012
*m*	0.017			0.012		

## Data Availability

The original contributions presented in the study are included in the article, further inquiries can be directed to the corresponding author.
